# Optimal location for gesture decoding in the sensorimotor cortex and implications for brain-computer interface research

**DOI:** 10.1016/j.neuroimage.2026.121837

**Published:** 2026-03-02

**Authors:** Maria Kromm, Mariana P Branco, Mathijs Raemaekers, Nick F Ramsey

**Affiliations:** a University Medical Center Utrecht Brain Center, Department of Neurology and Neurosurgery, Utrecht, the Netherlands; b Donders Institute for Brain, Cognition and Behaviour, Radboud University, Nijmegen, the Netherlands

**Keywords:** Brain-computer interfaces, High-field fMRI, Hand gestures, Classification

## Abstract

Implantable brain-computer interfaces (iBCIs) aim to restore communication in individuals with severe motor impairments. For good iBCI performance, it is important to target an optimal location. In this study, we used high-resolution 7-Tesla functional magnetic resonance imaging (fMRI) to map the spatial distribution of brain activity that can discriminate between a large number of hand gestures. Ten able-bodied participants performed 20 different unimanual hand gestures. Using support vector machines, we measured decodability across the cortex. The highest decoding performance was achieved in the hand region of the sensorimotor cortex. Moreover, we found that a subset of six well-distinguishable gestures could predict the optimal decoding location for the full set, suggesting that a carefully chosen subset can effectively guide pre-implantation mapping. Furthermore, while significant decoding was possible from sulcal as well as gyral regions of the precentral cortex, our analyses revealed that the sulcal area did not contribute unique information beyond that found in adjacent gyral regions. Similarly, decoding in the postcentral cortex was primarily driven by the gyrus. This indicates that surface recordings may suffice for iBCIs. Together, these findings offer practical guidance for future iBCI electrode placement, with the potential to improve communication and autonomy for individuals with severe motor impairments.

## 1. Introduction

Implanted brain-computer interfaces (iBCIs) aim to create a means of communication by establishing a pathway between signals measured directly from the brain and a computer (Wolpaw, 2013). This technology can potentially benefit individuals in a locked-in state, a neurological condition characterized by complete paralysis of voluntary muscles while being fully conscious. A locked-in state can result from, for example, Amyotrophic Lateral Sclerosis (ALS) or brainstem stroke (Laureys et al., 2005). The sensorimotor cortex (SMC) is assumed to be the ideal target location for iBCIs, as it has a detailed somatotopic organization (Dechent and Frahm, 2003; Penfield and Boldrey, 1937; Schellekens et al., 2018) and its activity corresponds directly to interactions with the environment (Georgopoulos et al., 1982; Morrow and Miller, 2003).

Within the hand region of the SMC, the precentral cortex is associated with hand motor control (Yousry et al., 1997), while the postcentral cortex processes mostly tactile and proprioceptive information (Stringer et al., 2014; Martuzzi et al., 2014). The SMC hand region serves as a suitable target for decoding both individual finger movements (Shen et al., 2014; Hotson et al., 2016; Wang et al., 2009), as well as more complex hand gestures (Bleichner et al., 2016, 2014; Bruurmijn et al., 2021). However, while this region likely constitutes a promising location for decoding hand movements, additional cortical areas may include alternative target locations (Gallego et al., 2022; Guan et al., 2023; Li et al., 2022; Ottenhoff et al., 2023). Identifying the locations with the highest decoding performance is essential for optimizing electrode placement in iBCIs.

Functional magnetic resonance imaging (fMRI) offers a non-invasive method for measuring neural activity with high spatial resolution, making it a valuable tool for identifying optimal electrode locations for iBCIs (Hermes et al., 2012; Leinders et al., 2023; Piantoni et al., 2021a; Ramsey et al., 2006; Van Den Boom et al., 2021a; Vansteensel et al., 2022). For example, previous studies have demonstrated that fMRI-based measures correlate highly with electrocorticography (ECoG) signals (Hermes et al., 2012; Siero et al., 2014), suggesting their utility in predicting the areas with optimal decoding performance. However, when using fMRI for pre-implantation mapping, only a limited set of movements can be tested due to time constraints. Thus, it is important to understand whether the cortical activity of a strategically chosen limited set of movements can be representative of a large range of movements.

While many iBCIs record mostly activity close to the cortical surface due to their placement on the gyri (Collinger et al., 2013; Hochberg et al., 2006; Metzger et al., 2023; Vansteensel et al., 2016; Willett et al., 2021), it is still unclear whether recordings from the sulcus could further improve BCI performance. fMRI can measure activity from both sulcal and gyral regions and can thus inform us on the potential contributions of the sulcus to hand movement decoding.

In this study, we measure neural activity during 20 different unimanual hand gestures in ten able-bodied participants using 7-Tesla fMRI. We focus on three objectives, including (1) identifying cortical areas with the highest decoding performance for hand gestures, (2) determining whether a small subset of distinguishable gestures can predict the optimal decoding location for a more extensive set of gestures, and (3) evaluating the contribution of sulcal versus gyral regions in decoding hand gestures. By examining these aspects, we aim to provide valuable insights to improve future electrode placement for gesture-based iBCIs, ultimately enhancing communication capabilities for individuals with severe motor impairments.

## 2. Methods

### 2.1. Participants

Ten able-bodied volunteers (age: *mean* = 25.5 years, *range* = 21 – 42 years; 6 females; all right-handed) were included in the study. All volunteers gave written informed consent to participate, and the Medical Research Ethics Committee of the University Medical Center Utrecht approved all experimental procedures according to the Declaration of Helsinki (2013).

### 2.2. Data acquisition

Images were acquired using a Philips Achieva 7 Tesla MRI system (Philips Healthcare, Best, The Netherlands) with a 32-channel receive head coil (Nova Medical, MA, USA). Functional volumes were recorded using an EPI pulse sequence (TR/TE = 1400/29 ms, flip angle (FA) = 60°, multiband factor 2, voxel size = 1.5 × 1.5 × 1.5 mm^3^, field of view = 200 × 200 mm^2^, 40 slices) with online correction for B0-induced distortions (EPIC). A high-resolution T1-weighted MP2RAGE (Marques et al., 2010) was acquired for anatomical reference (TE = 2.69 ms, FA = 6°, voxel size = 0.8 × 0.8 × 0.8 mm^3^, field of view = 200 × 200 mm^2^, 250 slices).

### 2.3. Experimental task

**Fig. 1.**
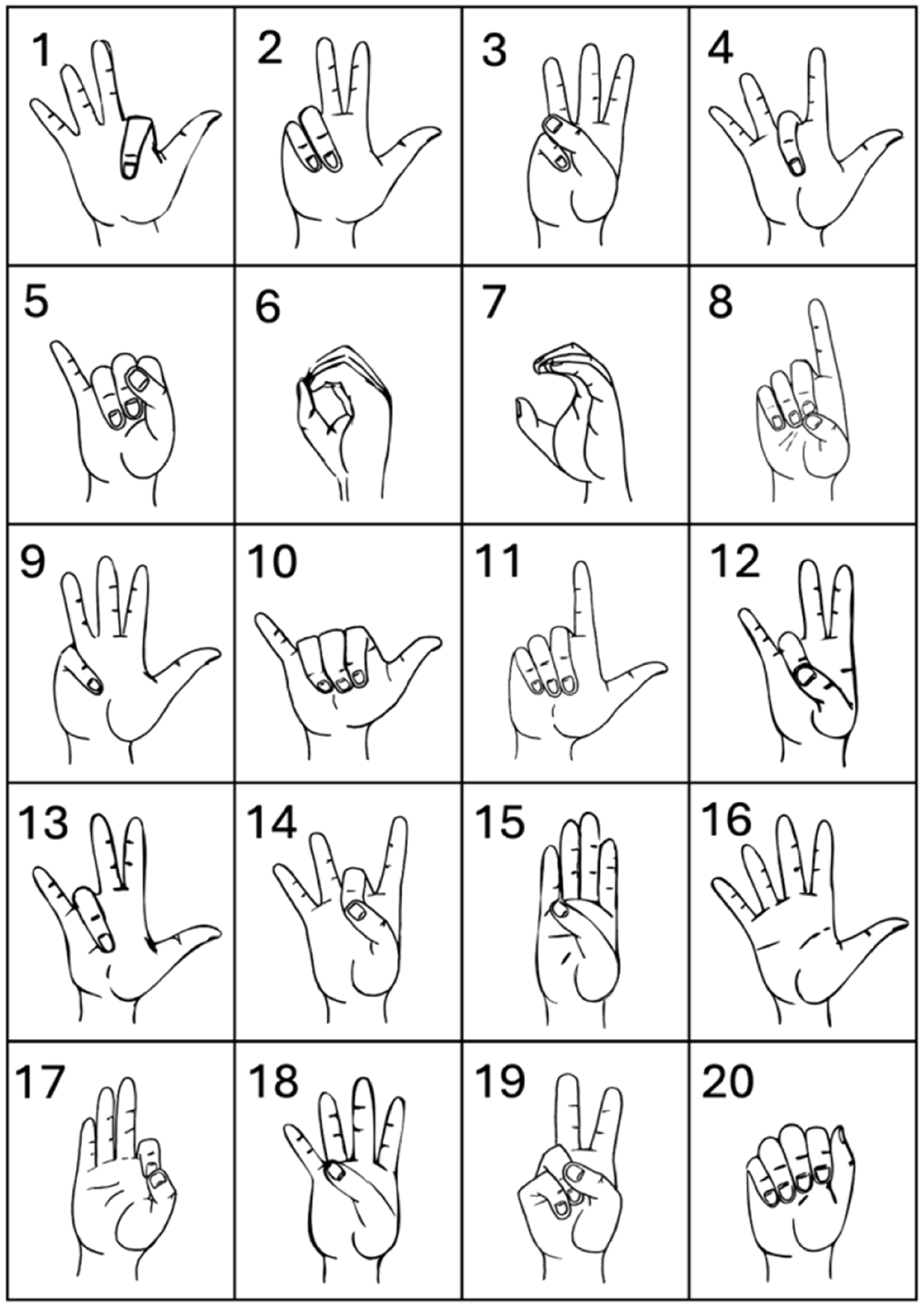
Gesture overview. The task contains 20 right-hand gestures, including 5 single-finger flexions (gestures “1″, “4″, “9″, “13″, “18”) and 15 gestures from the American Sign Language alphabet.

Participants performed the task with their right (dominant) hand. Fifteen gestures were selected from the American Sign Language alphabet based on ease of execution. In addition, we included individual flexion of each finger, resulting in a total of 20 gestures (Fig. 1). Participants received images of the hand gestures a week before scanning and were asked to practice the hand gestures at home to ensure familiarity with the movements.

**Fig. 2.**
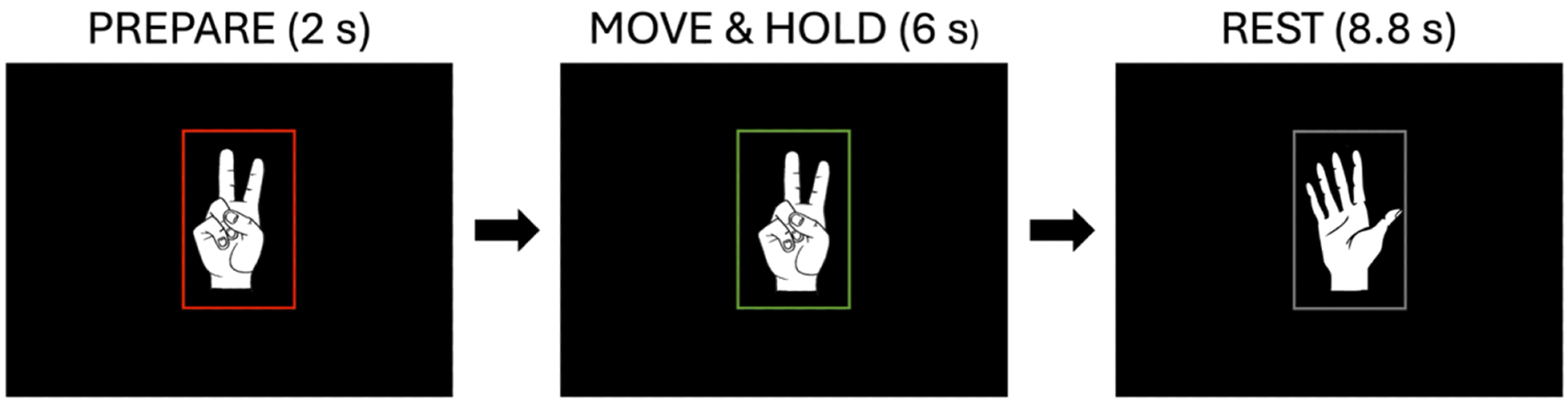
Task design. A trial consisted of three phases: 1) an image of a gesture inside a red rectangle signalled the onset of a preparation phase, which was included to minimize error in the execution of the movement; 2) the change of the rectangle’s color to green indicated to the participant that they should make the displayed gesture and hold it for the duration it was presented (move & hold phase); 3) after returning the hand position to baseline (hand relaxed, fingers slightly bent, palm face up), there was an 8.8 s resting period until the onset of the next stimulus (rest phase). The resting phase lasted for 8.8 s to prevent blood-oxygen-level-dependent (BOLD) responses from biasing the activity estimates of the subsequent trial.

The task was projected through a waveguide onto a screen mounted on top of the transmit coil. Participants viewed the screen through a mirror and prism glasses. Trials were presented in an event-related fMRI design (Fig. 2). The gestures were presented in a pseudo-random order, with each gesture performed once per run. Participants completed 10 runs in total, spread across two scanning sessions on separate days (5 runs/session). This yielded a total of 10 repetitions per gesture for each participant.

### 2.4. Task performance assessment

Behavioural data were recorded using MRI-compatible data gloves worn on both hands (5 DT Inc, Irvine, USA). To evaluate the task performance, we used a template matching approach on the data glove data of the right hand. Of all trials, 9.72 % were misclassified, meaning that the highest correlation was with a gesture template that differed from the one corresponding to the correct response. For the misclassified gestures, we then computed the ranking scores of the correlation with the correct templates. Of all misclassified trials, 95 % had a ranking score between 2 - 4, indicating that, in all likelihood, gestures similar to the correct gesture had been performed (Fig. S1). Given the low proportion of clearly incorrect trials, we decided to keep all trials for further analysis. Due to a technical issue, the data glove recording was missing for one participant (sub007); however, their fMRI data were still included in the main analysis because they did not show any unusual patterns.

### 2.5. Data preprocessing

**Fig. 3.**
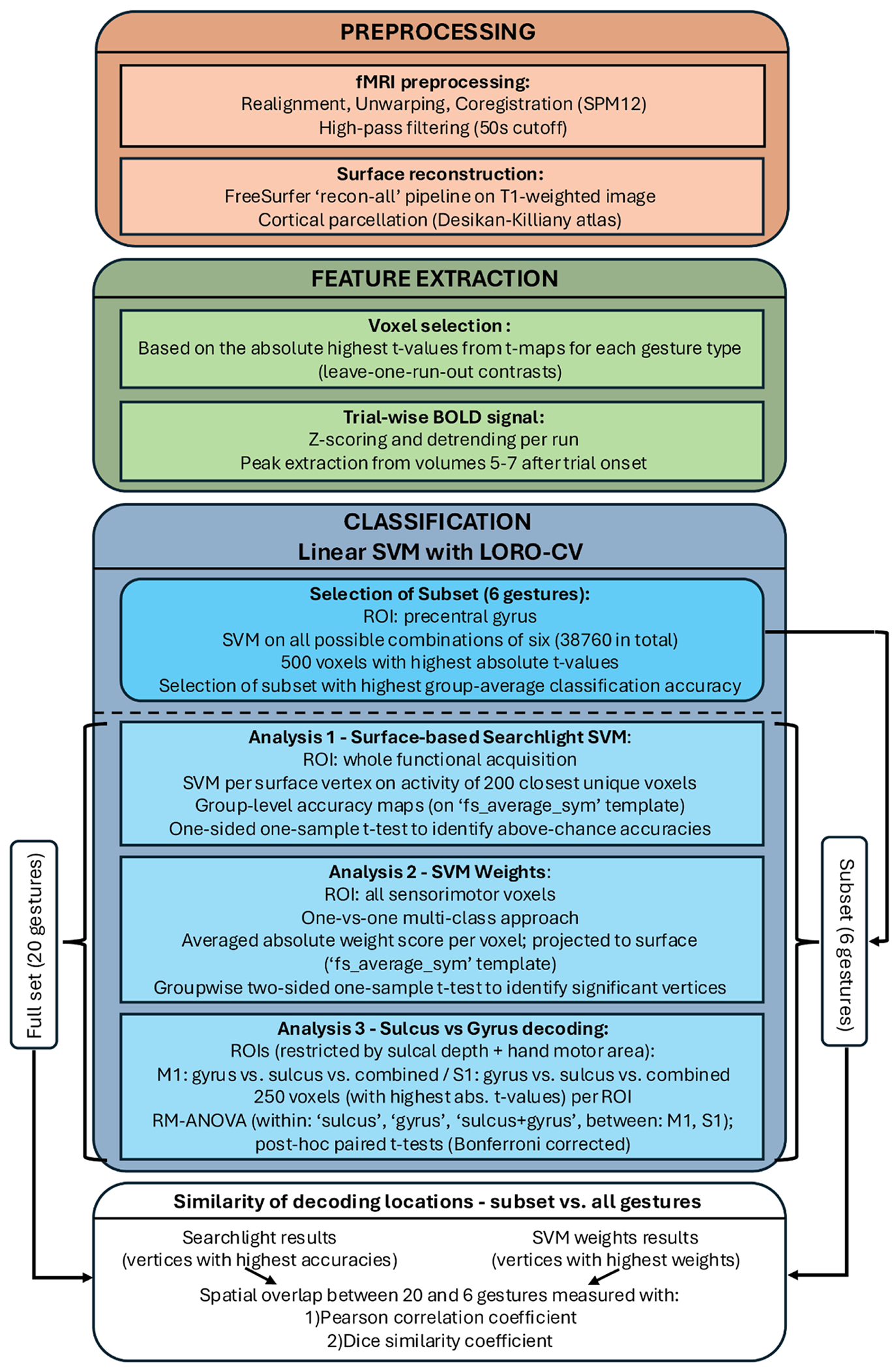
Overview of the analysis pipeline. SVM = Support Vector Machine. LORO—CV = leave-one-run-out cross-validation. ROI = region of interest. RM-ANOVA = repeated-measures ANOVA.

An overview of the full analysis pipeline can be seen in Fig. 3. Functional scans from the gesture task were pre-processed using SPM12 (http://www.fil.ion.ucl.ac.uk/spm/) and custom MATLAB^®^ (https://www.mathworks.com) scripts. Scans from both sessions were realigned, unwarped, and coregistered with the T1-weighted image. The resulting timeseries were high-pass filtered with a 50 s cut-off using a Gaussian-weighted running line filter.

A general linear model was created to estimate the blood-oxygen-level-dependent (BOLD) signal, including factors for each gesture. After estimation, *t*-maps were created representing activity for each gesture type (see section Gesture classification – general procedure), while using a leave-one-run-out procedure. This resulted in a total of 200 *t*-maps (20 gestures x 10 folds). FreeSurfer (Fischl, 2012) ‘recon-all’ pipeline was used to reconstruct the cortical surface based on the T1-weighted image. The resulting surface reconstructions were superimposed on a mean functional volume, and visually inspected to confirm the absence of geometric distortions or errors in image registration.

### 2.6. Regions of interest

All analyses only included the left hemisphere, which is the hemisphere contralateral to the moved hand. The regions of interest were defined using the FreeSurfer parcellation scheme that was based on the Desikan-Killiany atlas (Desikan et al., 2006).

### 2.7. Gesture classification – general procedure

To decode hand gestures, we used a linear support vector machine (SVM) classifier, treating the activity of each voxel as a separate feature. For data preparation, the timeseries from each voxel were detrended and transformed into z-scores within each run separately to remove any run-specific offsets. For each trial, the peak signal in the 5th, 6th, and 7th volumes after trial onset was extracted, corresponding to the amplitude of the peak of the BOLD response (Bleichner et al., 2014; Bruurmijn et al., 2021).

The SVM was run with a linear kernel and constraint parameter *C* = 1. To avoid dependencies between the training and testing datasets, we used a leave-one-run-out cross-validation scheme (LORO–CV). One run was left out from the training dataset in each fold, and the left-out run was used to test the model. This procedure was performed independently for each participant, meaning that training and testing were conducted within subjects (i.e., intra-subject cross-validation). Classification accuracy was computed as the proportion of correctly classified gestures for each fold, and the scores were then averaged to obtain a single classification score per participant. This procedure was repeated several times in each subject, using different subsets of voxels corresponding to distinct regions of interest and different sets of gestures, as described below. The number of voxels (i.e., features) varied depending on the region of interest.

### 2.8. Feature selection

In all analyses that selected features based on *t*-values, the selection was based on the training folds of the leave-one-run-out cross-validation. For fold *f, t*-contrasts were estimated based on activity maps from all runs except the left-out run*f*. Voxels were then ranked by absolute *t*-value from the leave-one-run-out *t*-contrasts, and the top *k* voxels (as specified per analysis) were selected as features. The classifier was trained on these features and evaluated for the left-out run *f*.

### 2.9. Selection of a subset of six well-distinguishable gestures

To investigate whether a smaller set of gestures can predict the optimal location for decoding all 20 gestures, we first identified a subset of six well-distinguishable gestures. We chose a set size of six as that is the minimum required for basic navigation (i.e., up, down, left, right, select, escape) (Kromm et al., 2024). Well-distinguishable gestures were chosen to allow for statistically powerful detection of discriminative brain activity when using a more limited set of gestures.

To identify the optimal set, we trained and tested the SVM on all possible combinations of six gestures (38,760 in total). Since these results should be informative for BCI-implants in individuals with locked-in syndrome, where sensory feedback is putatively absent (e.g., Rubio et al., 2022; Yu et al., 2022), we restricted the area used for training during this procedure to the precentral gyrus (M1). Specifically, we selected the 500 voxels with the highest absolute *t*-values across all gestures within the precentral gyrus as features. This procedure resulted in 38,760 classification accuracies per participant, each corresponding to one possible subset. The subset with the highest classification accuracy averaged over subjects was selected. Previous work has shown that subsets selected based on group-level performance tend to generalise well to individual-level decoding (Kromm et al., 2024).

To assess whether the M1-based subset also generalizes to the postcentral gyrus (S1), we repeated the full training and testing procedure restricted to S1, resulting in 38,760 classification accuracies specific to S1. To evaluate how well the M1-based subset performed in S1 relative to all other possible subsets, we calculated its percentile rank within the S1-based accuracy distribution for each participant.

### 2.10. Optimal decoding location

To identify brain regions that contain the most informative activity patterns for distinguishing between hand gestures, we applied two complementary analyses: (1) a searchlight SVM to map classification accuracy across the cortical surface, and (2) an SVM weights analysis to assess the contribution of each voxel to classification performance. These methods allowed us to compare the classification performance across the cortex and identify areas within the sensorimotor cortex that contribute the most to gesture decoding.

### 2.11. Searchlight

Assuming that areas other than the sensorimotor cortex may allow for decoding, we identified the areas with the highest classification accuracy across all cortical regions of the left hemisphere that were included in our functional acquisition. To this end, we performed a surface-based searchlight SVM analysis (Chen et al., 2011; Kriegeskorte et al., 2006). This approach allowed for a spatial identification of areas with high classification performance.

First, we mapped the volumetric pre-processed functional data to the cortical surface using an enclosing voxel algorithm, sampling at a 0.7 fraction of the grey matter thickness from the white matter surface. For each surface vertex, an SVM was trained and tested using the activity of the 200 closest unique voxels. Duplicate vertices mapping to the same voxel were removed by retaining only the first occurrence, ensuring consistent training data size. The analysis resulted in a classification accuracy per vertex.

For group-level analysis, the single-subject searchlight maps were registered to a standardized symmetrical surface template (‘fs_aver-age_*sym*’) (Greve et al., 2013) and smoothed with a 6 mm full-width half-maximum (FWHM) Gaussian kernel. A one-sided one-sample *t*-test was used to identify vertices with accuracy significantly above the theoretical chance level (*p* < 0.05; FWE corrected). The searchlight analysis was performed twice – once for all 20 gestures and once for the selected subset of six well-distinguishable gestures (for procedure, see ‘Selection of a subset of six well-distinguishable gestures’).

### 2.12. SVM weights

To examine the spatial distribution of the important discriminative features specifically over the sensorimotor cortex, we trained an SVM using all sensorimotor voxels as input features. Since our multi-class classification was implemented using a one-vs-one scheme, it yielded 190 binary classifiers (i.e., one for each unique pair among the 20 gestures) and 15 binary classifiers for 6 gestures across all sessions. From each of these binary SVMs, we extracted the weight vector (beta values) and computed the average of the absolute weights across classifiers and sessions to obtain a single weight score per voxel.

To project these scores to the cortical surface, the highest weight scores across grey matter layers were mapped to the surface, and results were subsequently registered to the standardized symmetrical surface template, with a Gaussian smoothing kernel of 6 mm FWHM.

For contrasting areas of high and low importance, we normalised the weights to z-scores for each subject. A groupwise two-sided one-sample *t*-test was used to identify the vertices with normalised weights significantly above or below zero. Vertices with high positive z-scores indicate locations of above-average contribution to decoding, while negative z-scores reflect below-average contributions. Similarly to the searchlight, the analysis of the SVM weights was performed twice – once for all 20 gestures and once for the selected subset of six well-distinguishable gestures (for procedure, see ‘Selection of a subset of six well-distinguishable gestures’).

### 2.13. Similarity of decoding locations - subset vs. all gestures

To assess how well the optimal decoding locations for the selected subset aligned with those for all 20 gestures, we estimated the Pearson correlation coefficient (Benesty et al., 2009) for the searchlight results and the SVM weights distributions. To confirm that a potentially high similarity is not driven by non-significant vertices, we estimated the Dice similarity coefficient (DSC) (Zou et al., 2004), a widely used measure of spatial overlap. We calculated the DSC for the top 1 %, 2 %, 5 %, 10 %, 15 %, and 20 % of all vertices. For the searchlight results, the top vertices were defined as those with the highest classification accuracies, while for the SVM weights, the top vertices reflected the highest normalised weight-values. The multi-threshold approach allowed us to assess the robustness of the spatial overlap across different inclusion thresholds.

To assess the spatial consistency of the brain areas important for decoding across individuals, we estimated the inter-subject variability by computing the weighted centroid of the top 1 % of searchlight accuracies and SVM-weights for each participant and measuring its Euclidean distance to the group-level centroid. In addition, we quantified the spatial similarity of the individual subject-level searchlight and SVM weights maps for both the 20 gestures and the subset of six gestures using Pearson correlation coefficients.

### 2.14. Contribution of the sulcus to gesture decoding

To evaluate the differential contribution of gyral and sulcal information, we assessed classification performance in six ROIs: ‘M1 gyrus’, ‘M1 sulcus’, ‘M1 gyrus+sulcus’, ‘S1 gyrus’, S1 sulcus’, and ‘S1 gyrus+sulcus’. These ROIs were defined using a combination of sulcal depth per voxel as assessed in the FreeSurfer surface reconstruction pipeline, and an ROI of the motor hand area based on an existing fMRI data set (van den Berg et al., 2025). The inclusion of voxels was restricted to the hand area to avoid confounding from activity differences associated with non-sensorimotor processes. The sulcal depth measure from the FreeSurfer pipeline assigns positive values to voxels located within sulci and negative values to voxels on gyri (Fischl, 2012). Accordingly, we classified voxels with a sulcal depth greater than zero as part of the sulcal ROI, and those with a depth less than zero as part of the gyral ROI. The volumetric data were mapped to the surface at a single depth fraction (0.7 of cortical thickness; 0 = white, 1 = pial); so depth-dependent BOLD effects were held constant across sulcal and gyral labels.

For each of the six ROIs, the 250 voxels with the highest absolute *t*-values across all gestures were selected as features. The number of voxels was based on a previously observed optimum when classifying activity in the sensorimotor cortex (Bleichner et al., 2014; Bruurmijn et al., 2021). To estimate the chance level and the associated confidence intervals, the classifier was also trained on randomly permuted labels, repeating the procedure 1000 times to obtain a chance distribution (Combrisson and Jerbi, 2015). The empirical significance threshold was set at the 95th percentile of the chance distribution.

To compare how sulcal and gyral regions contribute to classification accuracy, we conducted a repeated-measures analysis of variance (rm-ANOVA) with ‘sulcus’, ‘gyrus’, and ‘sulcus+gyrus’ as within-subject factors and M1 and S1 as separate measures. Although M1 and S1 were defined within each subject, we analysed them separately because they are anatomically and functionally distinct regions (e.g., Ann Stringer et al., 2014; Martuzzi et al., 2014; Yousry et al., 1997)(e.g., Ann Stringer et al., 2014; Martuzzi et al., 2014; Yousry et al., 1997). For significant within-subject effects (*p* < 0.05), post-hoc paired *t*-tests were performed with Bonferroni correction for multiple comparisons (alpha/number of performed tests). These analysis steps were run separately for all 20 gestures and the selected subset of six gestures.

## 3. Results

### 3.1. Gesture decoding performance is highest in the sensorimotor hand region

The group analysis of the searchlight results revealed a cluster with the highest decoding accuracy in the sensorimotor cortex (*range* = 19 – 32 %; Fig. 5A), corresponding to the location of the motor hand area (Van den Berg et al., 2025), with a larger coverage on the postcentral gyrus (S1) than the precentral gyrus (M1). Above chance-level group classification was observed outside of this cluster as well (*range* = 9 – 15 %), for example, in the premotor and posterior parietal regions and the supplementary motor area. However, the accuracy was substantially lower for these areas, suggesting that the information necessary to discriminate gestures is primarily localized in the sensorimotor hand region. Subject-specific searchlight hotspots, defined as each participant’s peak-accuracy location, were located on average 3.6 ± 2.8 mm from the group centroid of peak locations (*range* = 0.7 - 8.8 mm), indicating highly consistent localization across subjects (for the individual searchlight maps, see Figure S2).

In line with the searchlight results, the highest SVM classifier weights were observed in the hand region (Fig. 6), showing that this area contains the most informative features for gesture decoding within the sensorimotor cortex. Interestingly, areas more medial and lateral to the hand region within the central sulcus consistently showed low normalised SVM weights across subjects. These findings suggest that, while the hand region is highly involved, the adjacent areas along the central sulcus contribute substantially less to gesture decoding. The centroids of the subject-specific weight maps were located on average 7.1 ± 5.1 mm (*range* = 1.1 – 15.0 mm) from the group centroid, consistent with the broader and more distributed nature of classifier weight patterns. However, we still expect the highest weights to be located in the hand region (for the individual weight maps, see Figure S3), and within the tolerances relevant for EcoG grid placements.

### 3.2. A subset of six well-distinguishable gestures can predict the optimal location for decoding 20 gestures

**Fig. 4.**
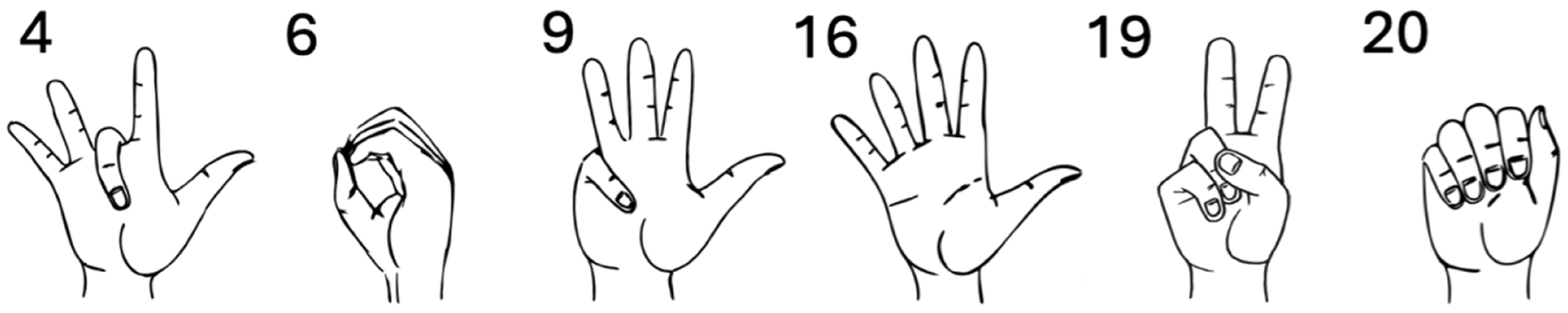
Best-performing gesture set. A subset of six gestures with the highest group-average decoding accuracy in M1.

To test whether a subset of six gestures can predict the optimal location for decoding a larger set of gestures, we first identified the set with the highest group-average decoding accuracy in the precentral cortex. The highest-performing set had a mean decoding accuracy of 65 % (*range* = 57 % - 80 %) and consisted of gestures with all-digit movements, wrist rotation, and various digit combinations (Fig. 4). This group-selected set shows a high performance for each subject in M1 (*mean percentile rank* = 96 %, *range* = 85.7 – 99.8 %; Fig. S4). Interestingly, this set also showed a high decoding performance in S1, ranking on average within the top 11 % of all possible subsets of six gestures (*mean percentile rank* = 89 %, *range* = 73 – 99 %) (Fig. S5). Thus, a set selected based on high performance in M1 is likely to also perform well in S1.

We then assessed the similarity of the optimal decoding locations of the selected set and those from all 20 gestures. We observed a high similarity for the patterns of classification accuracy (*Pearson r* = 0.98) and weights distribution (*Pearson r* = 0.96). This high similarity was confirmed in individual subjects for classification accuracy (*Pearson r range* = 0.91 – 0.94; Figure S2) and for the weights distribution (*Pearson r range* = 0.69 – 0.92; Figure S3)

**Fig. 5.**
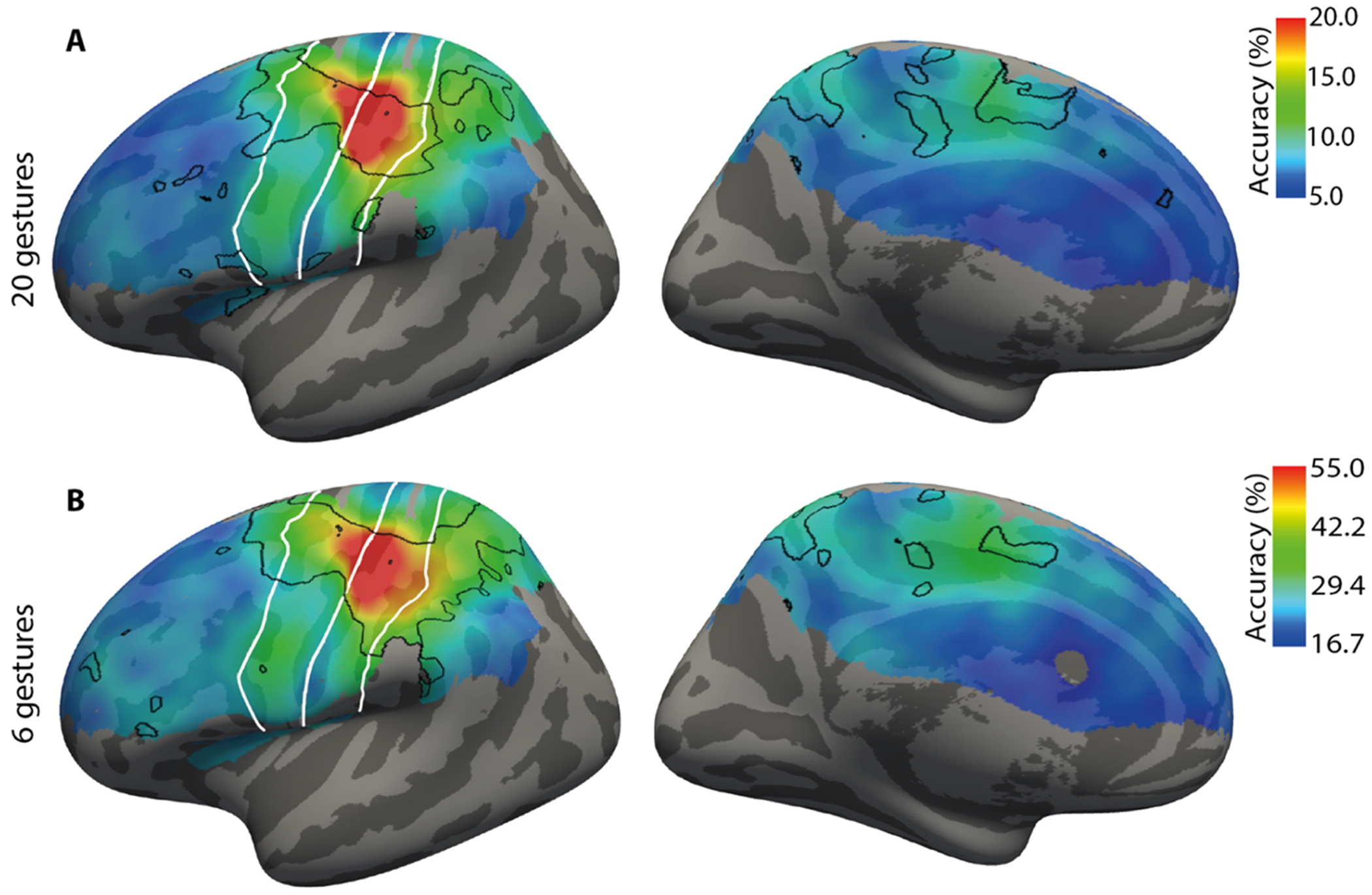
Searchlight classification results. Group mean (*n* = 10) results of the searchlight SVM analyses superimposed on the inflated surface of the symmetric fs_average template (left column: lateral view, right column: medial view). Classification accuracy of the searchlight for 20 gestures (A) and for the subset of six well-distinguishable gestures (B). Clusters of vertices where group-wise classification exceeded the chance level (alpha = 5 % corrected) are outlined in black. The white lines indicate the borders of the precentral and postcentral cortex. Vertices shown in grey were excluded from the group analysis because they fell outside the functional field of view in one or more participants. Only vertices with full coverage across all subjects were included.

**Fig. 6.**
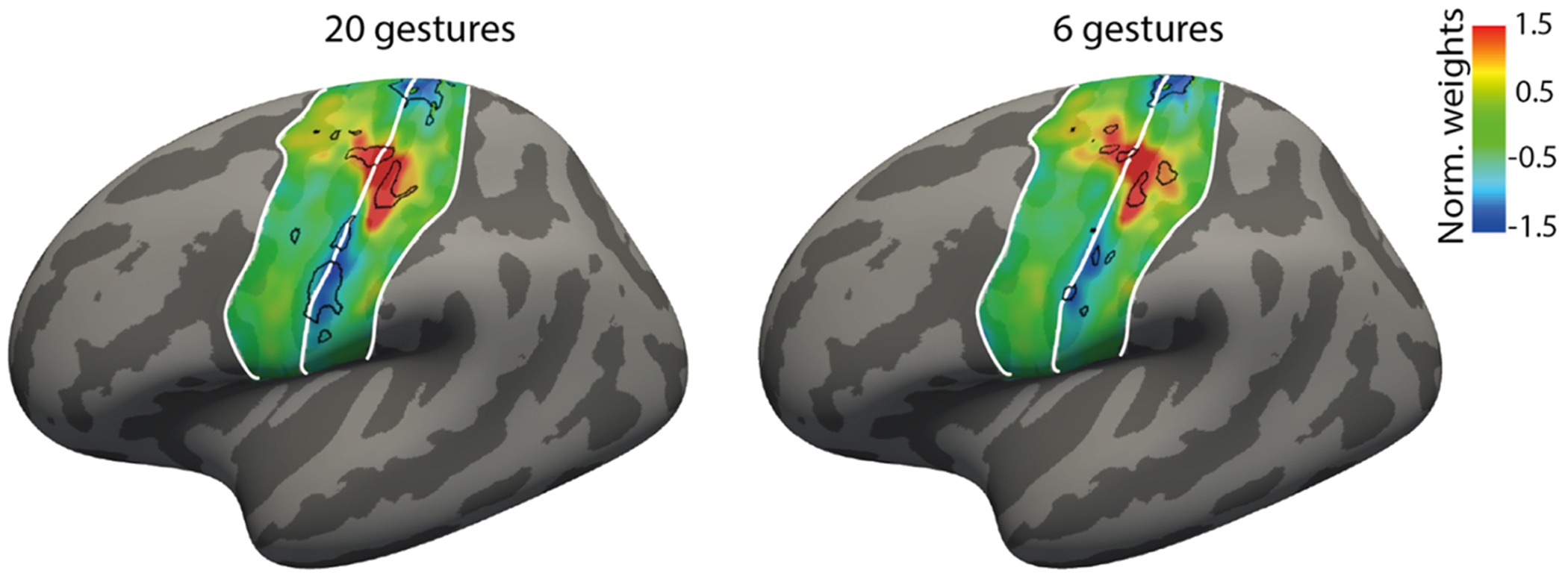
SVM-weights results. Group mean (*n* = 10) normalized SVM-weights results of the full sensorimotor cortex for decoding 20 gestures (left) and the subset of six gestures (right); superimposed on the inflated surface of the symmetric fs_average template. Clusters of vertices with normalized weights significantly higher or lower than zero are outlined in black. The white lines indicate the borders of the precentral and postcentral cortex.

In addition, the vertices with the highest classification accuracy strongly overlapped (for the top 1 %–20 % of vertices: *DSC_mean* = 0.92; *mean_range* = 0.9 – 0.95) (Fig. 5A–B), as did the locations with the highest SVM-weights (for the top 1 %–20 % of vertices: *DSC_mean* = 0.92; *mean_range* = 0.86 – 0.98) (Fig. 6), indicating that the locations deemed most important for decoding were nearly identical for the subset and all gestures.

### 3.3. All relevant decoding information can be found on the gyrus

The cluster with the highest classification accuracies and SVM weights also includes areas in the central sulcus, which are largely inaccessible by surface recordings. We therefore quantified the impact of not having access to this area.

**Fig. 7.**
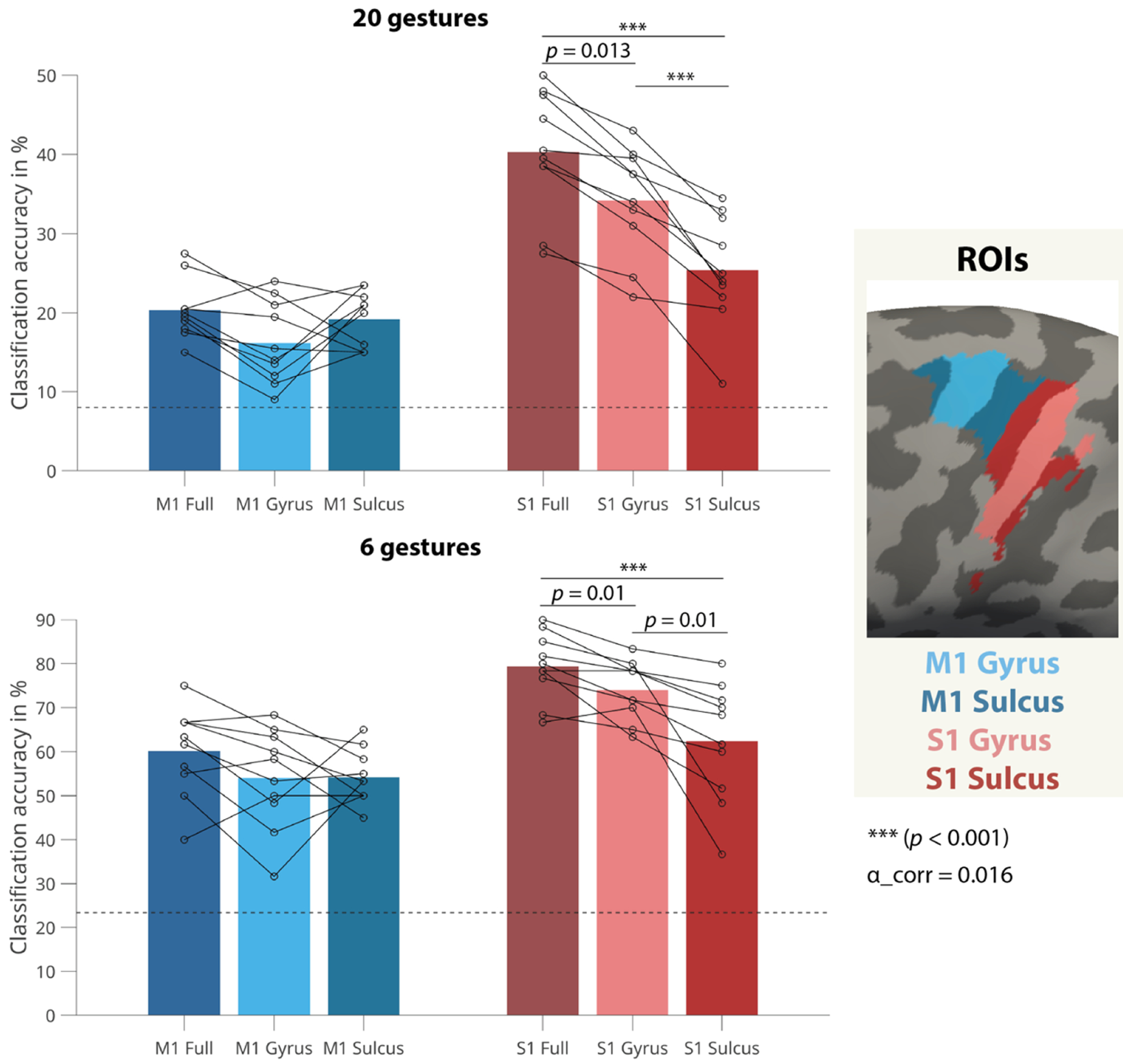
Classification in Sulcus vs Gyrus. Mean classification accuracy (*n* = 10) in M1 and S1 for sulcus, gyrus, and combined (‘full’) for 20 gestures (top) and the selected subset of six (bottom). The circles with black connecting lines represent the performances across different areas for each subject. The dashed black line indicates chance-level performance (for 20 gestures: 8 %; for the subset of six: 23.33 %). The features for classification were selected from the predefined regions of interest (ROIs) shown on the right.

In M1, classification accuracy did not differ significantly between the ‘M1 gyrus+sulcus’, ‘M1 gyrus’, and ‘M1 sulcus’ areas (Fig. 7). This was observed for both the full set of 20 gestures (*F*(2,18) = 1.82; *p* = 0.19) and the subset of six gestures (*F*(2,18) = 2.45; *p* = 0.12). There is thus no evidence that the sulcus provides additional information for gesture decoding in M1 beyond what is present on the gyrus.

In S1, however, classification accuracy differed significantly between the ‘sulcus’, ‘gyrus’, and ‘gyrus+sulcus’ (for 20 gestures: *F*(2,18) = 39.83; *p* < 0.001; for the subset: *F*(2,18) = 16.79; *p* < 0.001). The classification accuracy was significantly lower in ‘S1 sulcus’ compared to both ‘S1 gyrus’ (for 20 gestures: *t*(9) = 5.38, *p* < 0.001; for the subset:*t* (9) = 3.25, *p* = 0.01) and ‘S1 gyrus+sulcus’ (for 20 gestures: *t*(9) = 8.34, *p* < 0.001; for the subset:*t*(9) = 5.02, *p* < 0.001). Additionally, the accuracy for ‘S1 gyrus+sulcus’ was slightly higher than for ‘S1 gyrus’. Although this difference was statistically significant (for 20 gestures:*t*(9) = 2.66, *p* = 0.013; for the subset: *t*(9) = 3.28, *p* = 0.01), accounting for multiple comparisons (alpha = 0.016), the actual difference in accuracy scores was only 3 % for the 20 gestures and 5 % for the subset. These findings indicate that gesture decoding in S1 is primarily driven by gyral activity, with minimal additional contribution from the sulcus.

## 4. Discussion

In this study, we investigated the spatial distribution of information in the sensorimotor cortex necessary to discriminate between gestures to guide electrode placement for implantable brain-computer interfaces (iBCIs). Results from our SVM analyses demonstrate that the decoding performance of 20 hand gestures peaks on the hand region of the sensorimotor cortex. Moreover, we found that a strategically chosen subset of six well-distinguishable gestures was sufficient to predict the optimal decoding location for a broader set of 20 gestures. Crucially, while both sulcal and gyral regions contained decodable information, our analyses revealed that the sulcus in the precentral cortex did not contribute unique information beyond the gyrus, and decoding performance in the postcentral cortex was primarily driven by the gyrus.

### 4.1. Optimal location for gesture decoding

We found that most of the information necessary for discriminating between hand gestures is densely packed within the sensorimotor cortex, in the region that is associated with hand motor control (Yousry et al., 1997). Additional decodable information was observed in premotor and posterior parietal regions - areas where previous microelectrode array studies have demonstrated hand movement decoding (Andersen et al., 2019; Guan et al., 2023; Wandelt et al., 2022; Willett et al., 2020) – as well as medially in the supplementary motor area (SMA). These areas are involved in motor planning and higher-order aspects of motor control (Strick et al., 2021), like action timing (SMA; Lara et al., 2018; Russo et al., 2020), motor sequences (premotor cortex; Glaser et al., 2018; Yokoi and Diedrichsen, 2019), and sensorimotor integration (posterior parietal cortex; Cohen and Andersen, 2002). However, compared to the sensorimotor hand area, none of these areas showed clear decoding hotspots for discrete gestures in our data. Therefore, although they might complement sensorimotor signals in broader decoding applications (e.g., Gallego et al., 2022), our findings suggest that a constrained implant targeting the sensorimotor hand region is sufficient to capture a wide range of discrete hand movements.

### 4.2. Predicting the optimal location with a limited set of movements

For effective iBCI implantation, an ECoG grid should ideally be placed over cortical regions that support robust decoding across a wide range of hand actions. However, practical constraints during preoperative fMRI mapping limit the number of gestures that can be tested, compelling the use of more efficient strategies for identifying optimal electrode locations. To address this, we evaluated whether a small, well-chosen set of gestures could reliably predict the spatial decoding profile established by a wide range of movements. Our results show that a subset of six gestures with high individual decoding performance yielded an almost identical spatial pattern of optimal decoding as the full set of 20 gestures. We interpret this as evidence that well-decodable gestures likely involve a broad and differential engagement of muscle groups controlling the hand, thereby activating large portions of the sensorimotor hand area. This hypothesis is supported by the fact that several high-performing gesture subsets can also serve as effective proxies for full-set mapping, demonstrating substantial overlap among top-performing gesture combinations (Kromm et al., 2024)(Kromm et al., 2024). Moreover, the gesture subset that was selected based on performance in the precentral region also achieved high decoding accuracy in the postcentral region, suggesting a strong alignment in decodability patterns between these regions. This cross-regional generalizability indicates that gesture sets optimized for the precentral cortex may also be effective for the postcentral region, simplifying the mapping process.

### 4.3. Sulcal and gyral contributions to decoding

Implantable BCIs based on surface recordings such as electrocorticography (ECoG) have demonstrated the longevity and stability required for chronic implantation in iBCIs (Degenhart et al., 2016; Pels et al., 2019; Vansteensel et al., 2024; Volkova et al., 2019; Woeppel et al., 2021; Wyse-Sookoo et al., 2024), making the gyral cortex a practical target for long-term use. However, a substantial portion of the cerebral cortex lies within sulci (Ribas, 2010), prompting the question of whether deeper sulcal regions offer additional, functionally distinct information for decoding. Our study addressed this by systematically comparing gesture decoding performance from sulcal and gyral regions within the precentral and postcentral regions.

In the precentral cortex, decoding accuracy was comparable across the gyrus, sulcus, and their combination, indicating that the sulcal areas did not contribute unique information beyond what was already available from the gyrus. In the postcentral cortex, although decoding from sulcal voxels alone yielded lower performance, combining sulcal and gyral regions produced only modest gains compared to the gyrus alone. These findings differ from those of Yanagisawa and colleagues, who reported superior decoding performance from the sulcus relative to the gyrus (Yanagisawa et al., 2009). This discrepancy may stem from their use of clinical ECoG grids with 1 cm inter-electrode spacing, which could have limited the capture of discriminative signals depending on the spatial distribution of neural activity. In contrast, the 1.5 mm fMRI resolution employed in the current study enabled more comprehensive signal sampling. In addition, a recent preprint reported stronger sulcal segregation for limb-level representations (i.e., tongue, hand, foot) in fMRI (Deo et al., 2024). However, it is unclear whether this enhanced distinction in sulcal versus gyral representations would persist when going from individual limbs to the within-hand differential muscle engagement required by our gestures. To address this, electrophysiological recordings spanning both sulcal and gyral regions would be valuable.

Our results suggest that, at a millimetre-scale BOLD resolution, sulcal regions may share overlapping representational content with adjacent gyral areas, at least for the types of hand gestures investigated here. This has direct implications for iBCI design: surface-based recordings, which are largely restricted to gyral regions, may already capture the most informative neural signals for decoding hand movements. Moreover, this principle extends to other functional domains such as speech decoding, albeit with less pronounced effects (Guerreiro Fernandes et al., 2024), reinforcing the viability of high-performance iBCIs that rely solely on gyral recordings.

### 4.4. Limitations

A limitation of the current study is that the decoding results were observed in able-bodied participants performing executed movements, which could theoretically limit generalization to individuals with paralysis, who are the target users of iBCIs. Although some studies report weaker or noisier activation patterns following limb loss or spinal cord injury, it was demonstrated that somatotopic hand representations remain preserved (Kikkert et al., 2021, 2016). Together with evidence from intracortical recordings that movement-related responses follow a continuum (executed > attempted > imagined) (Dekleva et al., 2024; Rastogi et al., 2020), these findings suggest that activity patterns from individuals with motor impairment may be weaker compared to those of able-bodied controls but can still contain decodable, functionally meaningful information that can potentially be targeted by iBCIs.

Because the topography of hand representations remains intact after paralysis, the cortical regions identified here as optimal for decoding in able-bodied participants are likely to correspond to the same functional locations in individuals who rely on attempted movements. Supporting this, the sensorimotor cortex was shown to have stable somatotopy in paralysis (Shoham et al., 2001), decodability of attempted movements from intracortical and ECoG recordings (Hochberg et al., 2012; Wang et al., 2013), and decodability of complex hand gestures in amputees (Bruurmijn et al., 2017, 2021). Nevertheless, the reduced activity amplitude in paralysed individuals may lower the signal-to-noise ratio when discriminating gestures, so presurgical mapping may require additional trial repetitions compared to the six-gesture protocol validated in able-bodied participants. Future work directly comparing able-bodied and paralyzed participants can help quantify these effects and refine presurgical mapping procedures.

In addition, as individuals with paralysis would typically rely on attempted movements to control an iBCI system (Rubio et al., 2022; Yu et al., 2022), proprioceptive feedback would be absent, potentially reducing decoding performance in S1. We therefore based our decoding performance analysis on activity in the precentral gyrus. While it is important to acknowledge that M1 and S1 are strongly interconnected (Delhaye et al., 2018) and that the loss of sensory feedback could thereby theoretically impact activity in both regions, evidence in amputees (Bruurmijn et al., 2017; Kikkert et al., 2016) and spinal cord injury (SCI) (Kikkert et al., 2016, 2021) demonstrates that somatotopic hand representations persist in S1, even after a complete loss of sensory input. Click or tap here to enter text.This suggests that feedforward signals from M1 may contribute to maintaining representational structure in S1 despite sensory deprivation.

The absence of proprioceptive feedback may also affect the decodability to a different degree across sulcal and gyral portions of S1. Since the Brodmann area 3a (BA3a), located within the central sulcus, is a main receiver of proprioceptive input (Geyer et al., 1999; Sun et al., 2021), we would predict a larger reduction in sulcal relative to gyral decodability. However, as previously mentioned, the efferent signals from M1 might also result in sustained decodability across the whole S1. Our finding that surface-based recordings may capture the most informative signals for decoding should still hold during attempted movements (Cajigas et al., 2021; Bruurmijn et al., 2017, 2021).

Another potential limitation of our research is that decoding results are based on fMRI measures (i.e., BOLD response) while iBCIs are commonly driven by electrophysiological signals. It should be noted that our 1.5 mm fMRI voxels and typical ECoG contacts/pitches sample at (slightly) different spatial scales. Clinical grids use a ~10 mm pitch with an exposed conductive surface of ~2.4 mm, while HD grids use pitches of ~2–4 mm with ~1 mm exposed contacts. The ECoG-BOLD relationship was shown to be scale-dependent, showing the highest correlation when applying a 4 mm smoothing kernel to the fMRI data (Piantoni et al., 2021a, 2021b). Our fMRI results should be interpreted as identifying where decodable information is concentrated at a millimetre resolution. While previous work has demonstrated a high spatial correlation between the activity patterns of fMRI and ECoG (Hermes et al., 2012; Ojemann et al., 2013; Siero et al., 2014), the decoding performance with ECoG is likely to surpass that of fMRI due to the potential to access temporal movement information as well, which has been shown to enhance the decoding of hand movements (e.g., Branco et al., 2017)(e.g., Branco et al., 2017). As a result, our fMRI-based decoding may underestimate the potential performance achievable with ECoG.

Furthermore, observed effects of sulcal/gyral decoding may be the result of biases inherent to fMRI measurements. Firstly, fMRI signals originating from the M1 sulcus may be more susceptible to contamination from S1 signals due to misregistration or partial-volume effects than signals originating from the gyrus. We mitigated these effects with B0-distortion correction and native-space decoding using anatomy-guided ROIs (no surface smoothing). Importantly, if residual crosstalk/ partial volume between sulcal M1 and S1 were substantial, we would expect the M1 sulcus to benefit from signals in S1 (which showed overall higher decoding performance) and thus show an inflated decodability. This was not observed. Secondly, sulcal signals might be more affected by susceptibility-related signal reductions due to steeper brain tissue/CSF gradients in the sulcus compared to the gyrus. However, it is unlikely that this would explain the absence of a unique contribution of the sulcus to the decoding. If the sulcus were to contain unique decoding information, we would still expect to find that gyrus+sulcus would outperform gyrus alone, despite some sensitivity loss. This was not the case. We therefore interpret the lack of improvement for M1 gyrus+sulcus over M1 gyrus as evidence for largely overlapping, rather than unique sulcal information.

### 4.5. Recommendations for future BCI implants

Based on our findings, we can formulate several recommendations for the placement procedure of iBCIs based on discrete hand movements. We confirm that a well-defined region in the sensorimotor hand area can be used to decode up to 20 hand gestures. Moreover, we demonstrated that a small set of well-chosen, highly discriminable gestures, such as the subset presented here, can reliably predict the optimal decoding location, enabling efficient preoperative mapping with a limited number of movements. Our results also indicate that recordings from the gyrus capture most of the informative activity for decoding, while adding sulcal regions did not significantly enhance performance. Thus, surface cortical recordings may suffice for iBCI performance, at least at a millimetric spatial resolution (e.g., for ECoG recordings). Future work could build on these results by simulating electrode grid configurations to optimize electrode size, spacing, and placement, similar to the approach taken by Van den Boom and colleagues (Van Den Boom et al., 2021b)(Van Den Boom et al., 2021b)(Van Den Boom et al., 2021b)(Van Den Boom et al., 2021b). These recommendations are based on results obtained from able-bodied individuals who executed the hand movements, and future work should confirm these results in a clinical population.

## 5. Conclusion

In this study, we identified the optimal cortical location for decoding a wide range of unimanual hand gestures. Our findings show that the decoding performance was highest in a single area of the sensorimotor hand region, and that this area can be accurately identified using a small subset of six well-distinguishable gestures. Our results further demonstrate that including the central sulcus did not significantly enhance decoding performance over gyral regions alone, suggesting that surface electrodes might be sufficient to capture the most discriminatory information for gesture decoding at a millimetric spatial resolution. Together, these findings offer a foundation for future work in clinical populations to provide guidance for future implanted BCIs electrode placement, with the potential to improve communication and autonomy for individuals with severe motor impairments.

## Supplementary Material

1

Supplementary material associated with this article can be found, in the online version, at doi:10.1016/j.neuroimage.2026.121837.

## Data Availability

The data and code used during the current study are available from the corresponding author upon reasonable request.
