# Supplementary material for: Optimal location for gesture decoding in the sensorimotor cortex and implications for brain-computer interface research
Source: Neuroimage. Author manuscript; Available in PMC 2026 Jun 10. (PMC13249550; doi:10.1016/j.neuroimage.2026.121837)
Supplement: 1 [file NIHMS2179294-supplement-1.docx]

**Supplementary Materials**


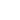

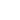


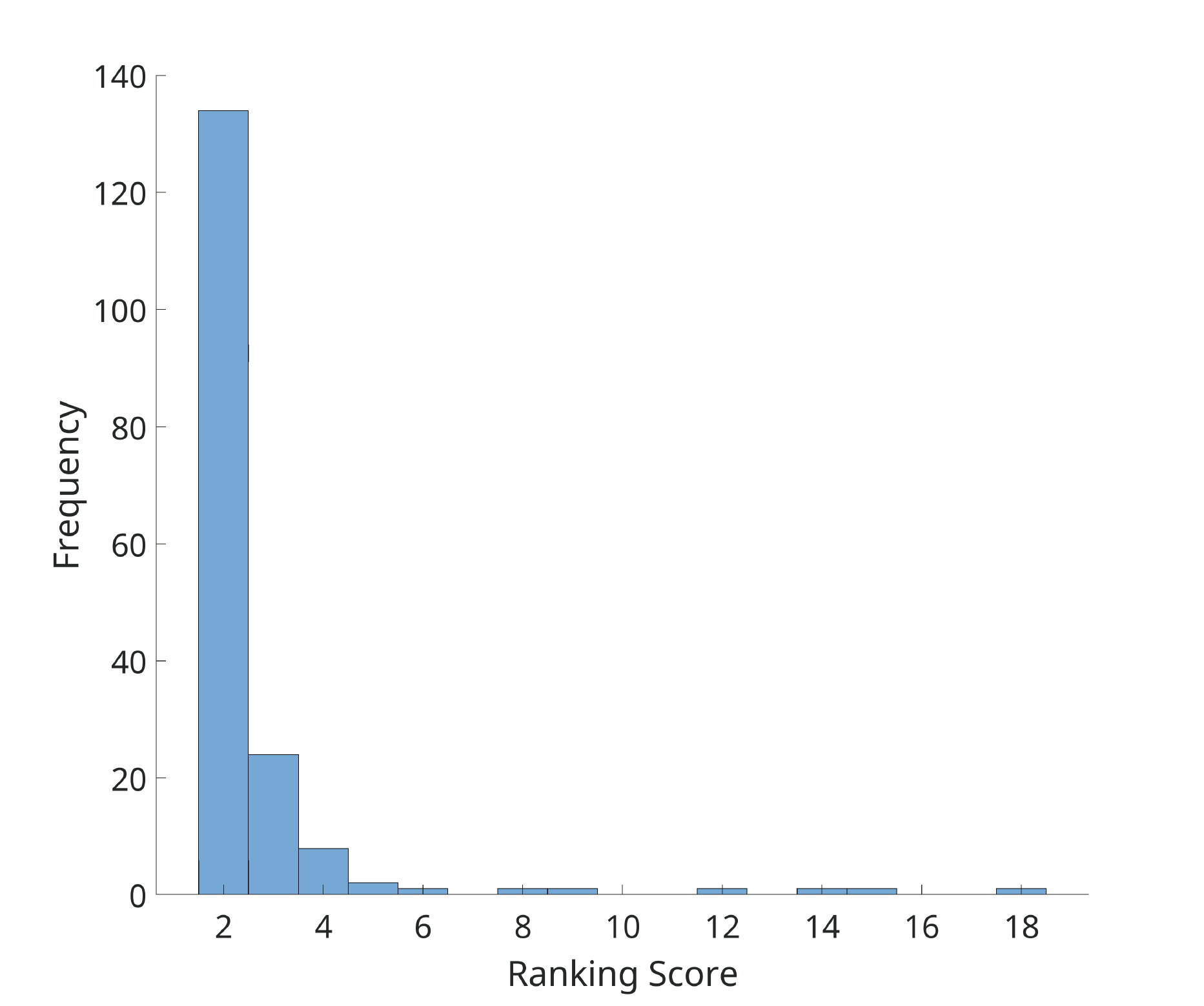


*Figure S1.* Data glove ranking scores of misclassified gestures (n = 9). Frequency distribution of ranking scores assigned to the true templates for incorrectly classified trials in the data glove data. A higher ranking score indicates lower similarity between the decoded gestures and the true template. Ranking score 1 (true template = decoded gesture) is not shown, as only misclassified gestures are taken into account.

*
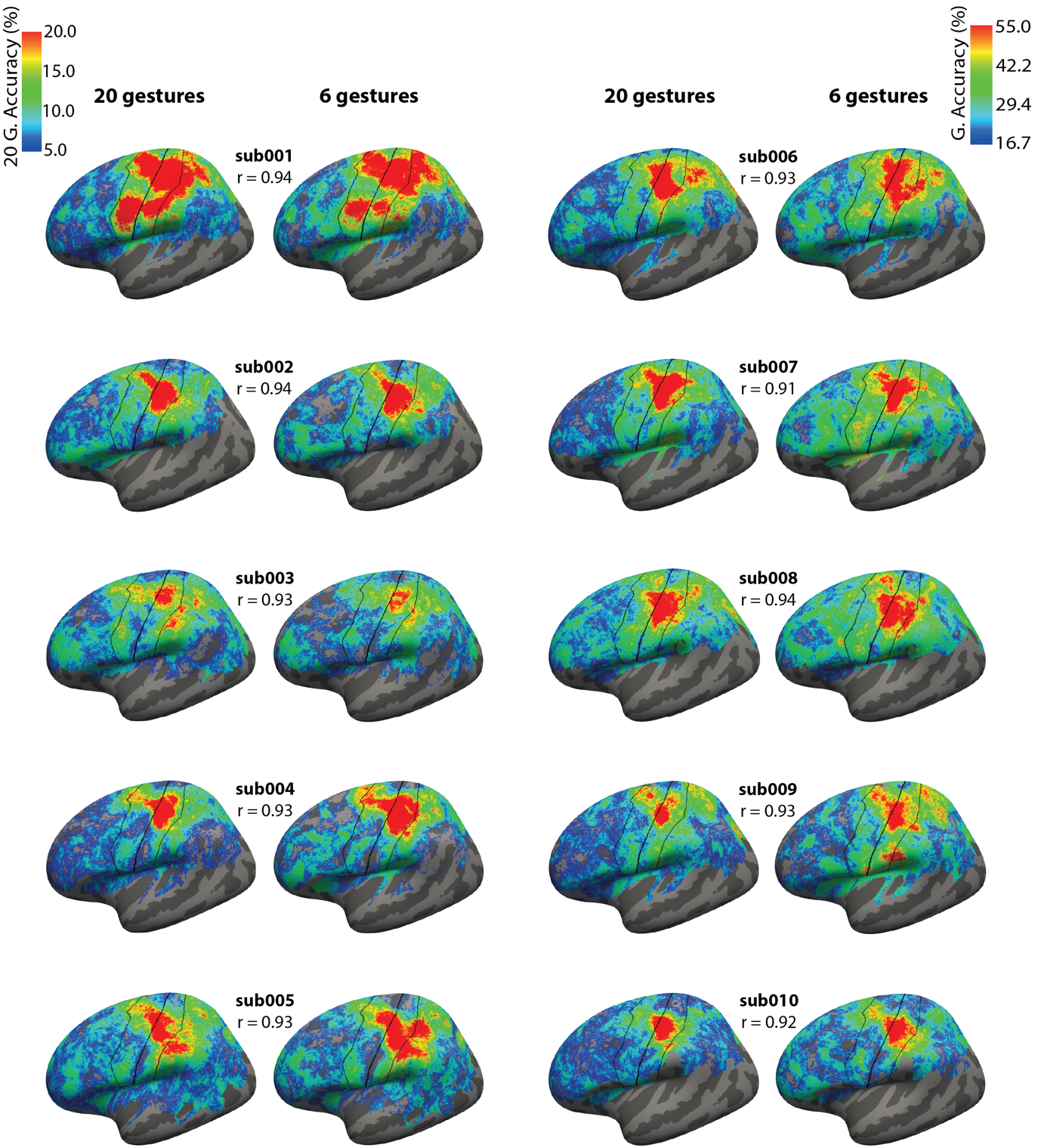
Figure S2*. Subject-specific searchlight classification results. Searchlight SVM classification accuracy maps for 20 gestures and for the subset of six well-distinguishable gestures for each participant, superimposed on the inflated surface of the symmetric fs_average template. The black lines indicate the borders of the precentral and postcentral cortex. For each participant, the accompanying r-value reflects the Pearson correlation between the 20‑gesture accuracy map and the 6‑gesture accuracy map, indexing the similarity between the two spatial patterns.


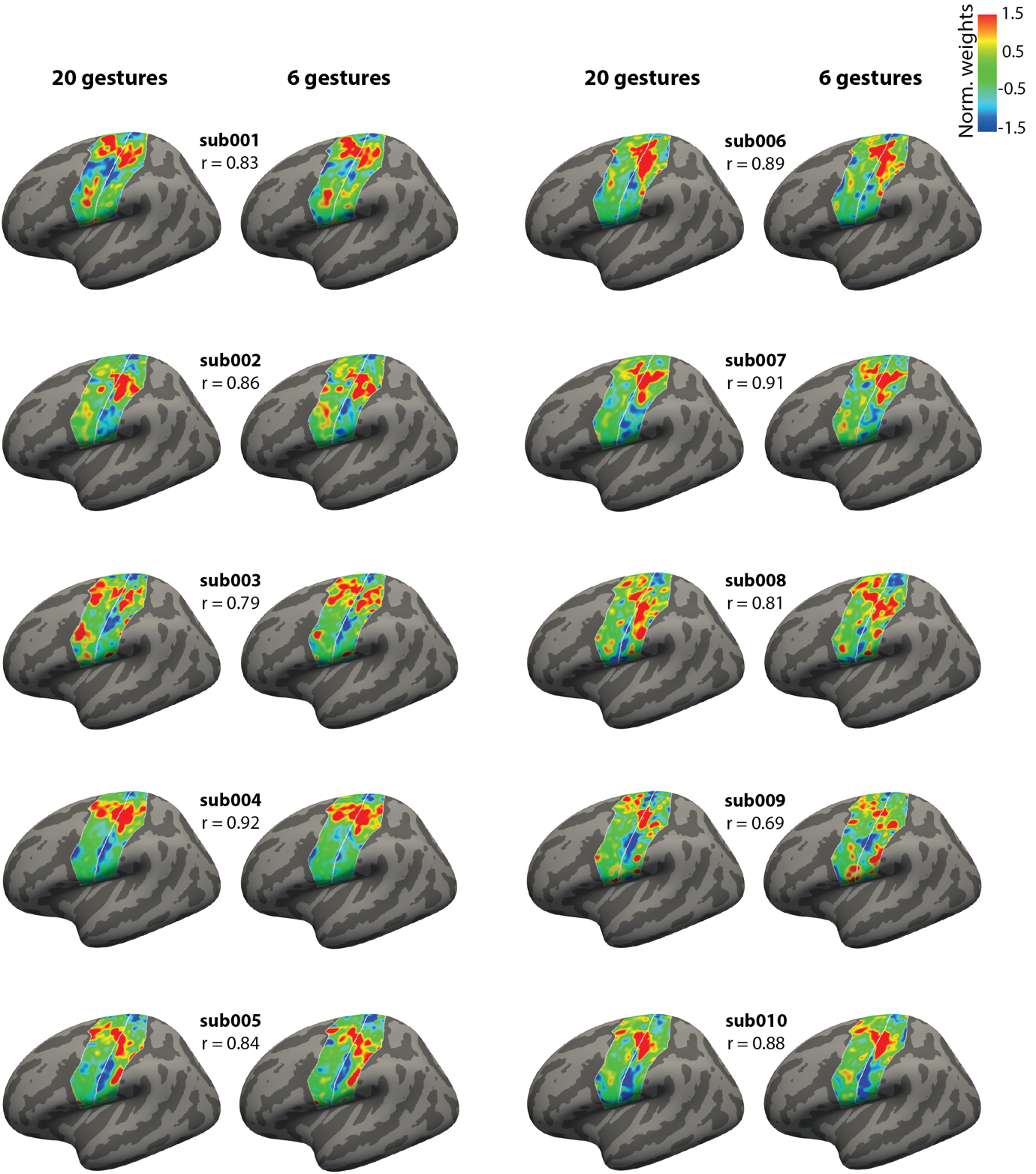


*Figure S3.* Subject-specific SVM‑weight maps. SVM-weight maps for 20 gestures and for the subset of six well-distinguishable gestures for each participant, displayed on the inflated surface of the symmetric fs_average template. The white lines indicate the borders of the precentral and postcentral cortex. For each participant, the accompanying *r*-value reflects the Pearson correlation between the 20‑gesture and 6‑gesture SVM‑weight maps, indexing the similarity of the weight distributions across gesture sets.


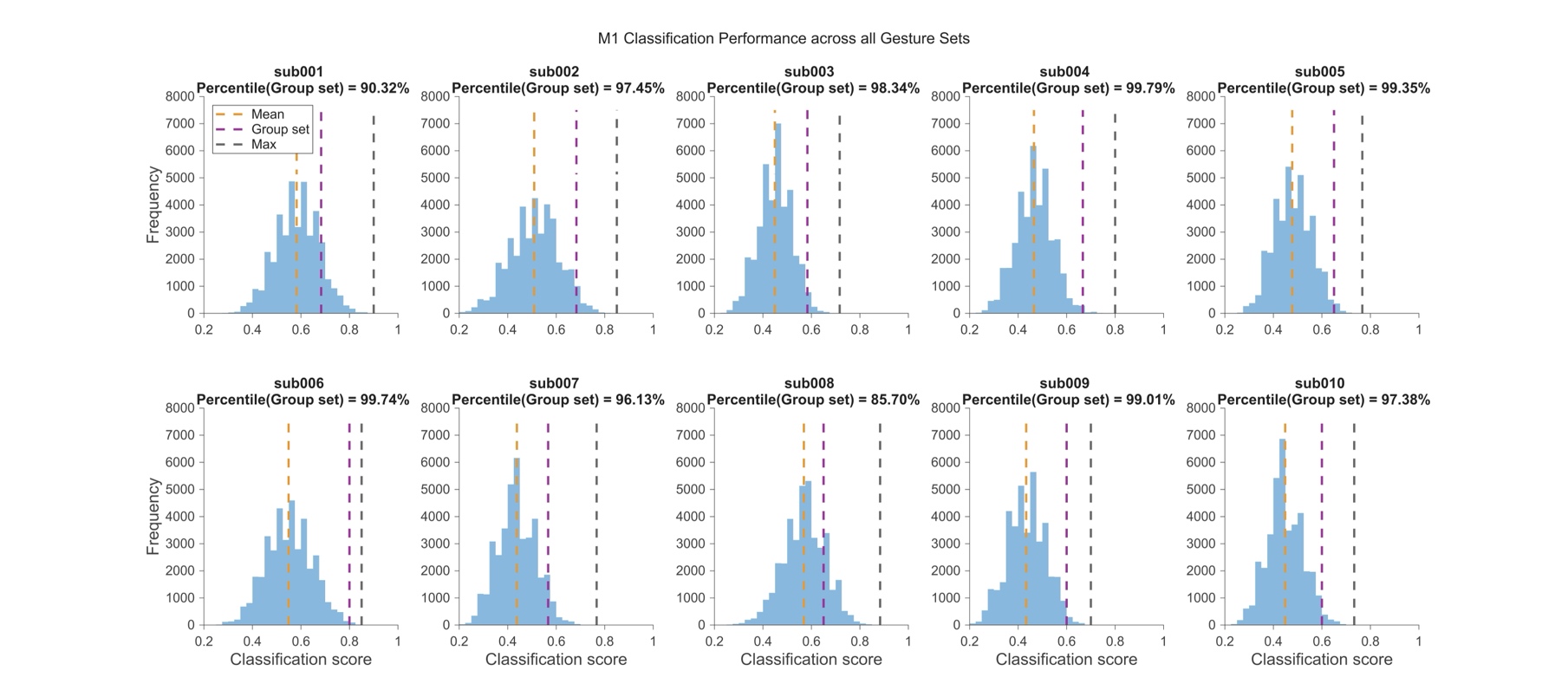


*Figure S4*. M1 classification performance across all gesture sets. Distribution of the classification performance across all subsets of 6 gestures (38760 combinations in total) in the precentral cortex (M1), per subject. The yellow line (“Mean”) indicates the average classification accuracy across all sets. The purple line (“Group set”) shows the accuracy of the selected gesture set based on the group average. The black line (“Max”) shows the highest classification score of the respective subject. The percentile score indicates how well the selected group set performs in M1 with respect to all the other sets for each participant.


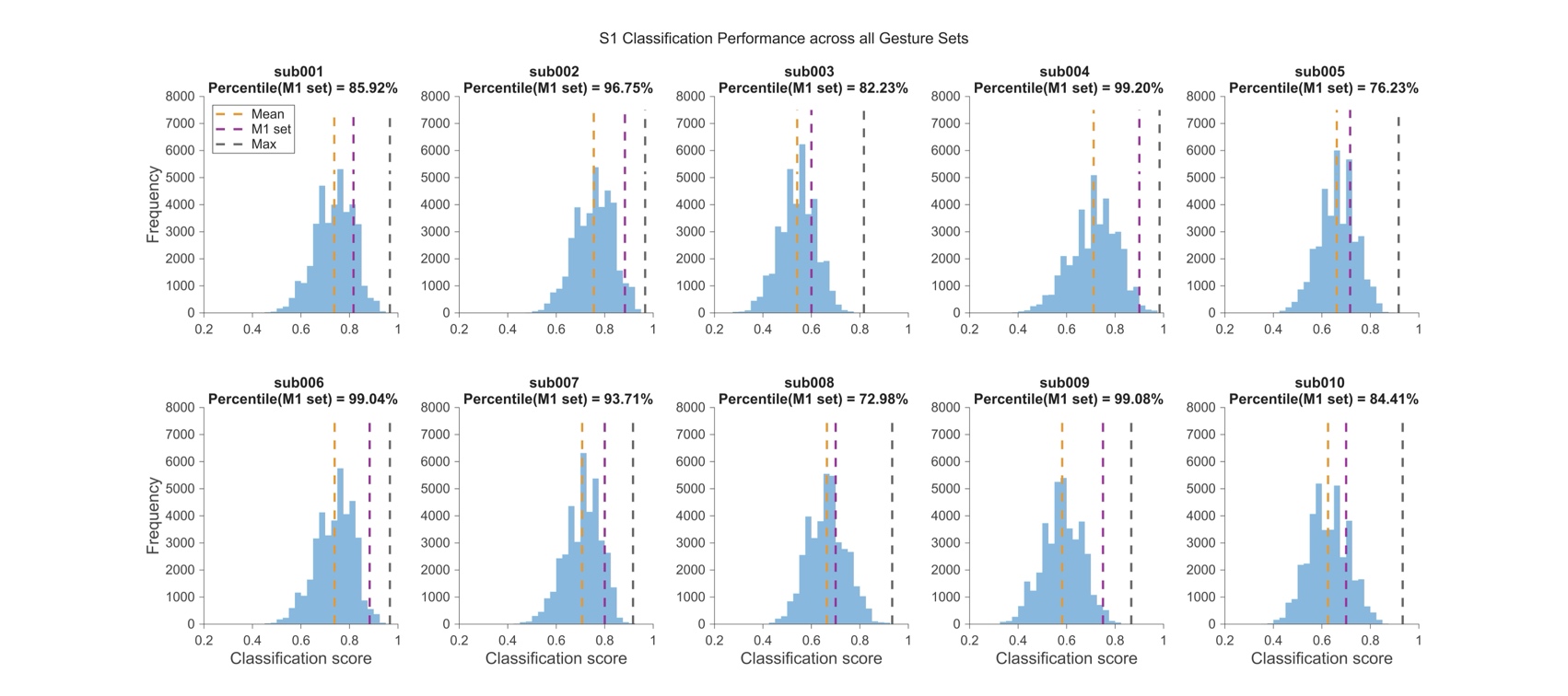


*Figure S5*. S1 classification performance across all gesture sets. Distribution of the classification performance across all subsets of 6 gestures (38760 combinations in total) in the postcentral cortex (S1), per subject. The yellow line (“Mean”) indicates the average classification accuracy across all sets. The purple line (“M1 set”) shows the accuracy of the selected gesture set from M1. The black line (“Max”) shows the highest classification score of the respective subject. The percentile score indicates how well the selected M1 set performs in S1 with respect to all the other sets.
